# The implementation of a required book club for medical students and faculty

**DOI:** 10.1080/10872981.2023.2173045

**Published:** 2023-01-30

**Authors:** David B. Ney, Nethra Ankam, Anita Wilson, John Spandorfer

**Affiliations:** a Department of Psychiatry Sidney Kimmel Medical College at Thomas Jefferson University, Philadelphia, PA; b The Department of Rehabilitation Medicine, Sidney Kimmel Medical College at Thomas Jefferson University, Philadelphia, PA; c Student Assessment Office, Sidney Kimmel Medical College at Thomas Jefferson University, Philadelphia, PA; d Sidney Kimmel Medical College at Thomas Jefferson University, Philadelphia, PA

**Keywords:** Medical students, narrative medicine, book club, social connection, wellness

## Abstract

More medical schools are incorporating wellness activities and the medical humanities into their curriculum. Finding implementable programming that is feasible and enjoyable is challenging. Both student participants and faculty who might facilitate programs are busy with clinical and educational responsibilities. Book club discussions in general are an activity that bring people together and expose groups to literature. In medical education, informal books clubs have been shown to increase camaraderie and expose participants to topics in medicine that they may not have encountered without the structure of the group assignment. At one large private urban medical school, all fourth year medical students were required to participate in a one-time hour-long book discussion with a faculty member one week before Match Day 2021. This paper describes the implementation of that program and discusses survey results from 179 students who broadly indicated that the books were enjoyable, the discussions were enriching, and that the program should continue for future classes of medical students.

## Introduction

In medicine, groups that are more engaged with their work perform better, have higher morale, and are more satisfied with their work [1]. Social connectedness, particularly spending time with colleagues in medicine, has been associated with mutual respect, collaboration, and appreciation for teammates [2–5].

One historically popular mechanism to spur social connection has been the ‘book club,’ wherein a book is pre-selected, read ahead of time, and discussed in a small group format. Group discussion can help readers uncover new opinions and refine previously held positions [6]. The often-informal group dynamic in a book club has its own benefits and has been linked to numerous positive outcomes, including the development of a professional identity, interprofessional communication, and bonding with colleagues [7–9].

In medical school, a book club can be used to introduce students to narrative medicine or to re-introduce the pleasure of reading. Narrative medicine, which includes the discussion of literature, encourages empathic imagination by putting the reader in the position of another and it facilitates knowledge acquisition by putting the reader in a new context that they might not be able to access through their own lived experience [10–12]. For medical students, reading and discussing literature enables growth and has been linked to increased communication skills as well as improvement in some empathy metrics [13,14].

In this paper, we evaluated the feasibility of a mandatory book club program that was implemented for the entire fourth year class of students at Sidney Kimmel Medical College (SKMC).

## Materials and methods

At a large private urban medical school, all fourth-year medical students were required to participate in a one-time hour-long book discussion with a faculty member in March 2021. The small groups were conducted virtually (via Zoom). Physician faculty were recruited based on their prior interest in the medical humanities and undergraduate medical education with some attention to specialty diversity. Faculty members selected the books that they would be discussing with some guidance from program developers (JS, NA) to avoid redundance and to encourage diverse options. In June 2020, students were provided the list of books with faculty facilitator name and specialty, and then selected one of 28 groups with a maximum of twelve students per group. Students were provided with physical copies of their respective books at the beginning of their fourth academic year and then discussions occurred in March just prior to Match Day while the students were participating in other class-wide didactic programming. Students were sent periodic reminders to read their books before the session. There were no writing or reflection assignments associated with the book – the only expectation was that students would complete the book and come prepared to discuss it with faculty and peers. Within the sessions, students were encouraged to share their broader impressions of the book and discuss lessons learned from their readings. Immediately after the book discussion, students anonymously evaluated the program with a Qualtrics survey, which was deemed to be exempt by the University Institutional Review Board.

The answers to all survey questions were strongly disagree, disagree, neither agree nor disagree, agree, or strongly agree. The questions included 1) the book choices were broad enough to encompass my interests, 2) I enjoyed reading the book, 3) the faculty facilitator was effective, 4) reading and discussing this book enriched my medical education, 5) Reading and discussing this book allowed me to reflect on an aspect of healthcare I did not previously consider, and 6) I would recommend that this program continue.

## Results

279 students choose one of 28 books (Table 1). Faculty facilitators represented twelve specialties: internal medicine (9), emergency medicine (3), pediatrics (3), surgery (3), family medicine (2), psychiatry (2), anesthesiology (1), neurology (1), neurosurgery (1), obstetrics-gynecology (1), ophthalmology (1), and physical medicine and rehabilitation (1).

**Table 1.** List of book titles with authors that students read and discussed.

Title	Author
A Leg to Stand On	Oliver Sacks
An Anthropologist on Mars	Oliver Sacks
Better	Atul Gawande
Black Man in a White Coat	Damon Tweedy
Complications	Atul Gawande
Confessions of an Anesthesiologist	William Cottrel
Cutting for Stone	Abraham Verghese
Do No Harm: Stories of Life, Death, and Brain Surgery	Henry Marsh
Heart: A History	Sandeep Jauhar
In Shock	Rana Awdish
Incidental Finding	Danielle Ofri
Island of the Color Blind	Oliver Sacks
Letters to a Young Doctor	Richard Selzer
Life after Loss	Vamik Volkan and Elizabeth Zintl
Mountains Beyond Mountains	Tracy Kidder
Out of My Life and Thought	Albert Schweizter
Rush	Stephen Fried
Salt in My Soul	Mallory Smith
Singular Intimacies, Becoming a Doctor at Bellevue	Danielle Ofri
The Cancer Journals	Audre Lorde
The Doctor Stories	William Carlos Williams
The Good Doctor	Barron Lerner
The Immortal Life of Henrietta Lacks	Rebecca Skloot
The Plague	Albert Camus
The Spirit Catches You and You Fall Down	Ann Fadiman
This Side of Doctoring: Reflections from Women in Medicine	Eliza Chin
Weekends at Bellevue	Julia Holland
When Breath Becomes Air	Paul Kalanithi

178 (63.8%) students responded to the survey. 88.3% somewhat or strongly agreed that the book choices were broad enough to encompass their interest. 82.8% somewhat or strongly agreed that they enjoyed reading the book. 98.3% somewhat or strongly agreed that their faculty facilitator was effective. 80.0% somewhat or strongly agreed that reading and discussing this book enriched their medical education. 77.8% somewhat or strongly agreed reading and discussing this book allowed them to reflect on an aspect of healthcare they did not previously consider. Finally, 77.2% somewhat or strongly agreed that they would recommend this program to continue in the curriculum.

Students were asked to comment about the program, broadly providing positive feedback:

*I thought there was a great variety of choices, plenty of time to read the book before I needed to discuss it, and the smaller group facilitated a nice discussion.*

*Diverse set of interesting book choices. Became talking points on the residency trail. Forced me to get into reading again.*

*I thought this was a great program, informal, no fast deadlines, no homework assignments, just a good book with [a] brief reflective journal club. I appreciated the simplicity of it.*

Others felt there should be additional sessions in the preclinical years or include an option to sign up for multiple book sessions:

*It would be nice to have the opportunity to read multiple books and attend multiple sessions.*

Several students requested that non-medical books be included and that the sessions not be mandatory:

*If this program continues, I recommend that books be changed to encompass non- medical books as well. We will be spending the vast majority of our time for the next 3–8 years in the hospital and after four years, an application cycle and the stress of match week, it was draining to read a book about the ethics of medicine*.

## Discussion

Our analysis, based on survey results from 178 students, indicates that the implementation of this program was effective, and the book discussions were enjoyed.

Our findings built upon work from Henderson et al who found that participation in an informal book club between medical students and faculty helped to cultivate strong mentor-mentee relationships. The activity helped to forge bonds between peers by creating shared activities outside of the typical curriculum [15]. This expanded upon previous work by Chisolm et al which found that informal book clubs amongst physicians fostered community and reduced feelings of burnout [16].

The use of virtual book club meeting and discussion may limit the generalizability of these findings and likely impacted the student experience of the project. Future iterations of this program could include different books and more frequent sessions for those who wish to participate more. Additionally, future surveys may focus less on overall enjoyment of the program and begin to assess more fully if and how the program may improve social connection, professional identity, or appreciation for the medical humanities.

The purpose of the book club was to bring students together to discuss medicine and literature in an informal setting. The book club project was well-received by nearly all surveyed students. While many medical schools are exploring immersive ways to inject the medical humanities into their curricula, this study proposes a simple but effective way to bridge those goals and deliver a popular activity that can be re-created for future classes.
